# Long-Term Outcomes and Genetic Predictors of Response to Metastasis-Directed Therapy Versus Observation in Oligometastatic Prostate Cancer: Analysis of STOMP and ORIOLE Trials

**DOI:** 10.1200/JCO.22.00644

**Published:** 2022-08-24

**Authors:** Matthew P. Deek, Kim Van der Eecken, Philip Sutera, Rebecca A. Deek, Valérie Fonteyne, Adrianna A. Mendes, Karel Decaestecker, Ana Ponce Kiess, Nicolaas Lumen, Ryan Phillips, Aurélie De Bruycker, Mark Mishra, Zaker Rana, Jason Molitoris, Bieke Lambert, Louke Delrue, Hailun Wang, Kathryn Lowe, Sofie Verbeke, Jo Van Dorpe, Renée Bultijnck, Geert Villeirs, Kathia De Man, Filip Ameye, Daniel Y. Song, Theodore DeWeese, Channing J. Paller, Felix Y. Feng, Alexander Wyatt, Kenneth J. Pienta, Maximillian Diehn, Soren M. Bentzen, Steven Joniau, Friedl Vanhaverbeke, Gert De Meerleer, Emmanuel S. Antonarakis, Tamara L. Lotan, Alejandro Berlin, Shankar Siva, Piet Ost, Phuoc T. Tran

**Affiliations:** ^1^ Department of Radiation Oncology, Rutgers Cancer Institute of New Jersey, Robert Wood Johnson Medical School, Rutgers University, New Brunswick, NJ; ^2^ Department of Radiation Oncology and Molecular Radiation Sciences, Johns Hopkins University School of Medicine, Baltimore, MD; ^3^ Department of Pathology and Human Structure and Repair, University of Ghent, Ghent, Belgium; ^4^ Department of Biostatistics, Epidemiology, and Informatics, Perelman School of Medicine, University of Pennsylvania, Philadelphia, PA; ^5^ Department of Radiation Oncology, Ghent University Hospital, Ghent, Belgium; ^6^ Department of Pathology, Johns Hopkins University School of Medicine, Baltimore, MD; ^7^ Department of Urology, Ghent University Hospital, Ghent, Belgium; ^8^ Department of Radiation Oncology, Mayo Clinic, Rochester, MN; ^9^ Department of Radiation Oncology, University of Maryland School of Medicine, Baltimore, MD; ^10^ Department of Radiology and Nuclear Medicine, Ghent University, and Department of Nuclear Medicine, AZ Maria-Middelares Ghent, Belgium; ^11^ Department of Radiology, Ghent University Hospital, Ghent, Belgium; ^12^ Department of Pathology, Ghent University Hospital, Ghent, Belgium; ^13^ Department of Nuclear Medicine, Ghent University Hospital, Ghent, Belgium; ^14^ Department of Urology, AZ Maria-Middelares Ghent, Ghent, Belgium; ^15^ Department of Oncology, Sidney Kimmel Comprehensive Cancer Center, Johns Hopkins University School of Medicine, Baltimore, MD; ^16^ Departments of Medicine, Urology and Radiation Oncology, UCSF, San Francisco, CA; ^17^ Department of Urologic Sciences, University of British Columbia, and Vancouver Prostate Centre, Vancouver, Canada; ^18^ James Buchanan Brady Urological Institute, Johns Hopkins School of Medicine, Baltimore, MD; ^19^ Department of Radiation Oncology, Stanford University School of Medicine, Stanford, CA; ^20^ Department of Epidemiology & Public Health, University of Maryland School of Medicine, Baltimore, MD; ^21^ Department of Urology, Catholic University Leuven, Leuven, Belgium; ^22^ Department of Urology, AZ Nikolaas, Sint-Niklaas, Belgium; ^23^ Department of Radiation Oncology, Catholic University Leuven, Leuven, Belgium; ^24^ Department of Medicine, University of Minnesota School of Medicine, Minneapolis, MN; ^25^ Department of Radiation Oncology, Princess Margaret Cancer Center, Toronto, Canada; ^26^ Department of Radiation Oncology, Peter MacCallum Cancer Center, Melbourne Australia; ^27^ Department of Radiation Oncology, Iridium Network, Antwerp, Belgium; ^28^ Department of Human Structure and Repair, Ghent University, Ghent, Belgium

## Abstract

*Clinical trials frequently include multiple end points that mature at different times. The initial report, typically based on the primary end point, may be published when key planned co‐primary or secondary analyses are not yet available. Clinical Trial Updates provide an opportunity to disseminate additional results from studies, published in* JCO *or elsewhere, for which the primary end point has already been reported.*

The initial STOMP and ORIOLE trial reports suggested that metastasis-directed therapy (MDT) in oligometastatic castration-sensitive prostate cancer (omCSPC) was associated with improved treatment outcomes. Here, we present long-term outcomes of MDT in omCSPC by pooling STOMP and ORIOLE and assess the ability of a high-risk mutational signature to risk stratify outcomes after MDT. The primary end point was progression-free survival (PFS) calculated using the Kaplan-Meier method. High-risk mutations were defined as pathogenic somatic mutations within *ATM*, *BRCA1*/*2*, *Rb1*, or *TP53*. The median follow-up for the whole group was 52.5 months. Median PFS was prolonged with MDT compared with observation (pooled hazard ratio [HR], 0.44; 95% CI, 0.29 to 0.66; *P* value < .001), with the largest benefit of MDT in patients with a high-risk mutation (HR high-risk, 0.05; HR no high-risk, 0.42; *P* value for interaction: .12). Within the MDT cohort, the PFS was 13.4 months in those without a high-risk mutation, compared with 7.5 months in those with a high-risk mutation (HR, 0.53; 95% CI, 0.25 to 1.11; *P* = .09). Long-term outcomes from the only two randomized trials in omCSPC suggest a sustained clinical benefit to MDT over observation. A high-risk mutational signature may help risk stratify treatment outcomes after MDT.

## INTRODUCTION

The use of metastasis-directed therapy (MDT) is rapidly increasing in the setting of oligometastasis. STOMP and ORIOLE, the only two prospective trials of stereotactic ablative radiation versus observation in metachronous oligometastatic castration-sensitive prostate cancer (omCSPC), demonstrated that MDT, as compared with observation, prolong androgen deprivation–free survival^1^ and progression-free survival (PFS).^2^ Although MDT appears to be effective in omCSPC, little is known regarding the utility of biomarkers to guide treatment for these patients.^3,4^ Thus, the goal of this study was to report long-term outcomes of STOMP and ORIOLE and assess the ability of genomics to stratify treatment response after MDT.

## METHODS

Comprehensive details regarding STOMP and ORIOLE have been reported previously.^1,2^ Both were prospective phase II trials enrolling individuals with omCSPC, defined as ≤ three metastases, with random assignment to observation or MDT. Active systemic therapies were not allowed with MDT. Both had institutional review board approval, and all participants provided informed consent.

Next-generation sequencing was performed on primary prostate tumor or blood from patients enrolled. A high-risk mutational signature was defined as pathogenic somatic mutations within *ATM*, *BRCA1*/*2*, *Rb1*, and *TP53* on the basis of their strong association with prostate cancer outcomes.^2-8^ Pathogenic mutations were defined by commercial tests and the publicly available COSMIC tumor variant database.^3^

The primary end point of interest was PFS as defined previously.^2^ Additional end points included radiographic progression-free survival (rPFS) defined as development of new nodal lesions, intrapelvic or distant, bone, or visceral lesions or death. Time-to-event analysis was performed to detect differences in end points of interest using the Kaplan-Meier method, stratified by treatment (MDT *v* observation) or high-risk mutational status. All analyses were conducted using R version 4.1.1.^9^

## RESULTS

### Clinical Outcomes After MDT

**FIG 1.**
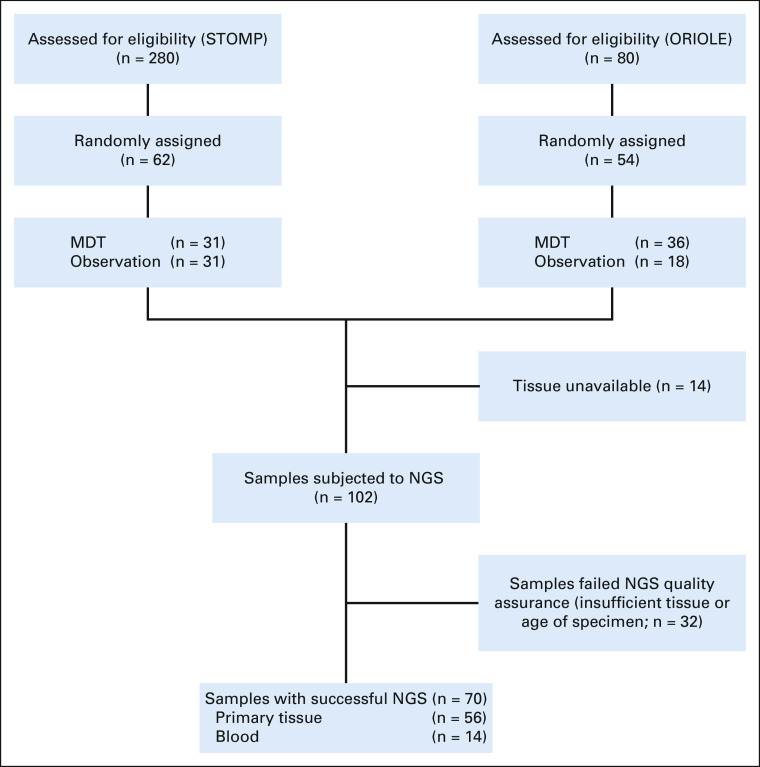
CONSORT diagram demonstrating screening, inclusion, and sequenced sample breakdown. MDT, metastasis-directed therapy; NGS, next-generation sequencing.

One hundred and sixteen patients in total were included for analysis—62 from STOMP and 54 patients from ORIOLE. The CONSORT diagram is shown in Figure 1. Baseline characteristics were well balanced between groups (Table 1). The median follow-up was 52.5 months (range, 5.8-92.0 months).

**TABLE 1.**
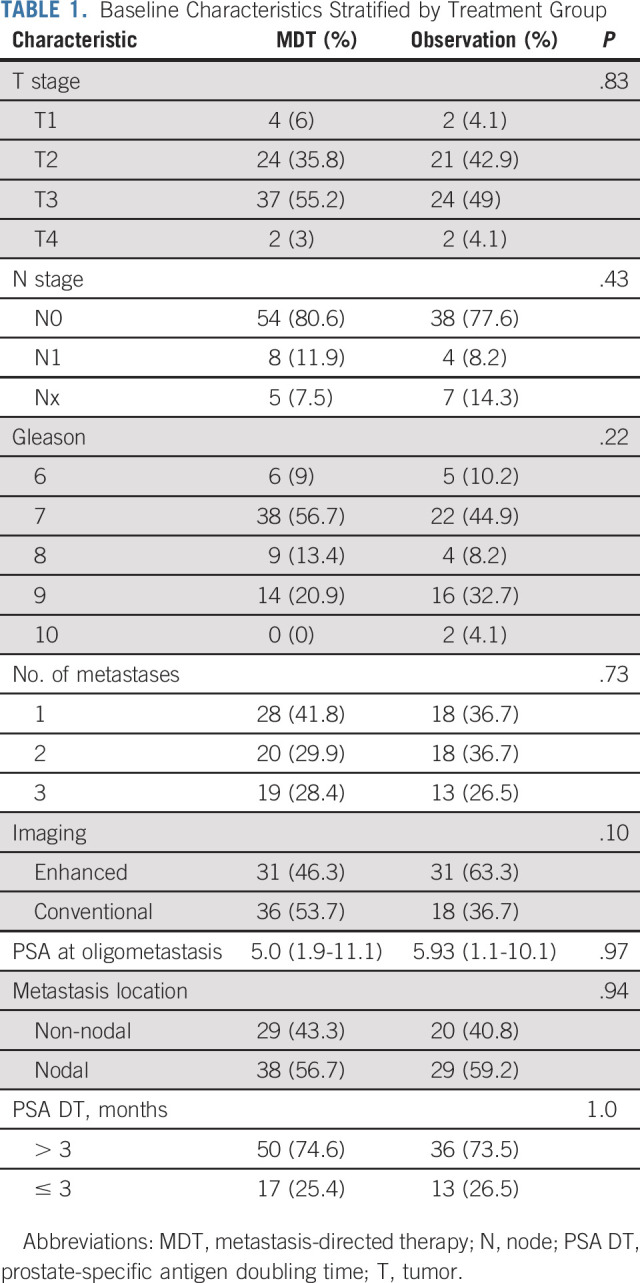
Baseline Characteristics Stratified by Treatment Group

**FIG 2.**
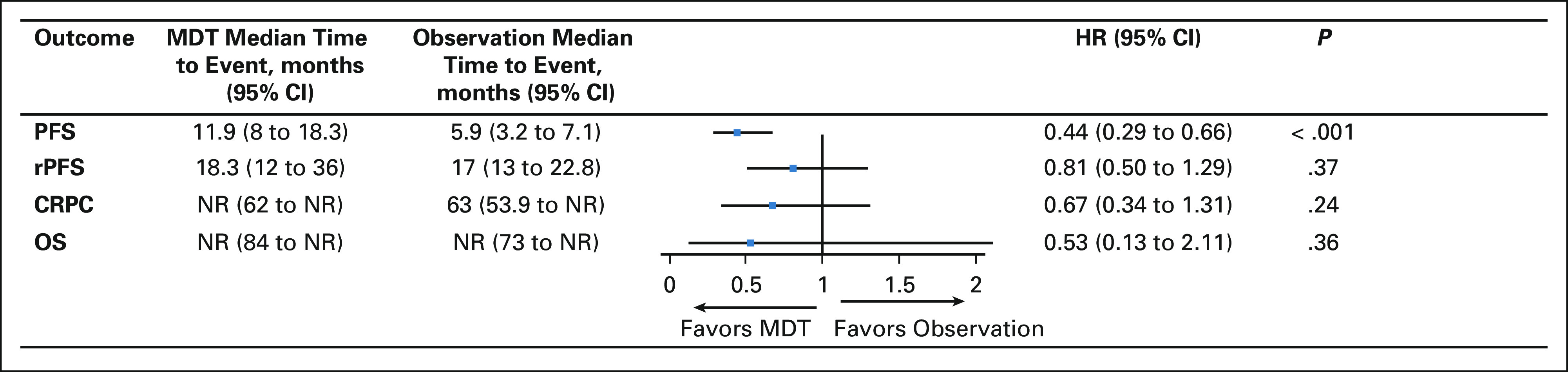
Time-to-event outcomes of MDT versus observation. Time-to-event outcomes demonstrate improvements in PFS with MDT over observation, but no differences in rPFS, time to CRPC, or OS. CRPC, castration-resistant prostate cancer; HR, hazard ratio; MDT, metastasis-directed therapy; NR, not reached; OS, overall survival; PFS, progression-free survival; rPFS, radiographic progression-free survival.

PFS was prolonged with MDT in both trials (Data Supplement, online only). The median PFS for the pooled cohort was 11.9 months (95% CI, 8.0 to 18.3) with MDT compared with 5.9 months (95% CI, 3.2 to 7.1) with observation. This corresponded with a pooled hazard ratio (HR) of 0.44 (95% CI, 0.29 to 0.66; *P* value < .001, Fig 2). The pooled HR for rPFS, time to castration-resistant prostate cancer, and overall survival did not differ between treatment groups (Fig 2 and Data Supplement).

### Genetic Features and Impact on Outcomes

A total of 103 patients (89%) had tissue available for sequencing, and 70 patients (60%) had tissue that was successfully subjected to somatic next-generation sequencing (Fig 1). Clinical characteristics of these 70 patients are given in the Data Supplement and are similar to the entire cohort. In the entire population, the median PFS in those without a high-risk mutation was 11.9 months (95% CI, 7.0 to 16.3) compared with 5.9 months (95% CI, 5.8 to 11.1) in those with a high-risk mutation (HR, 0.57; 95% CI, 0.32 to 1.03; *P* = .06, Data Supplement). In those without a high-risk mutation, the median rPFS was 22.6 months (95% CI, 18.1 to 36) compared with 10.0 months (95% CI, 5.9 to 17.1) in those with a high-risk mutation (HR, 0.38; 95% CI, 0.20 to 0.17; *P* < .01, Data Supplement).

**FIG 3.**
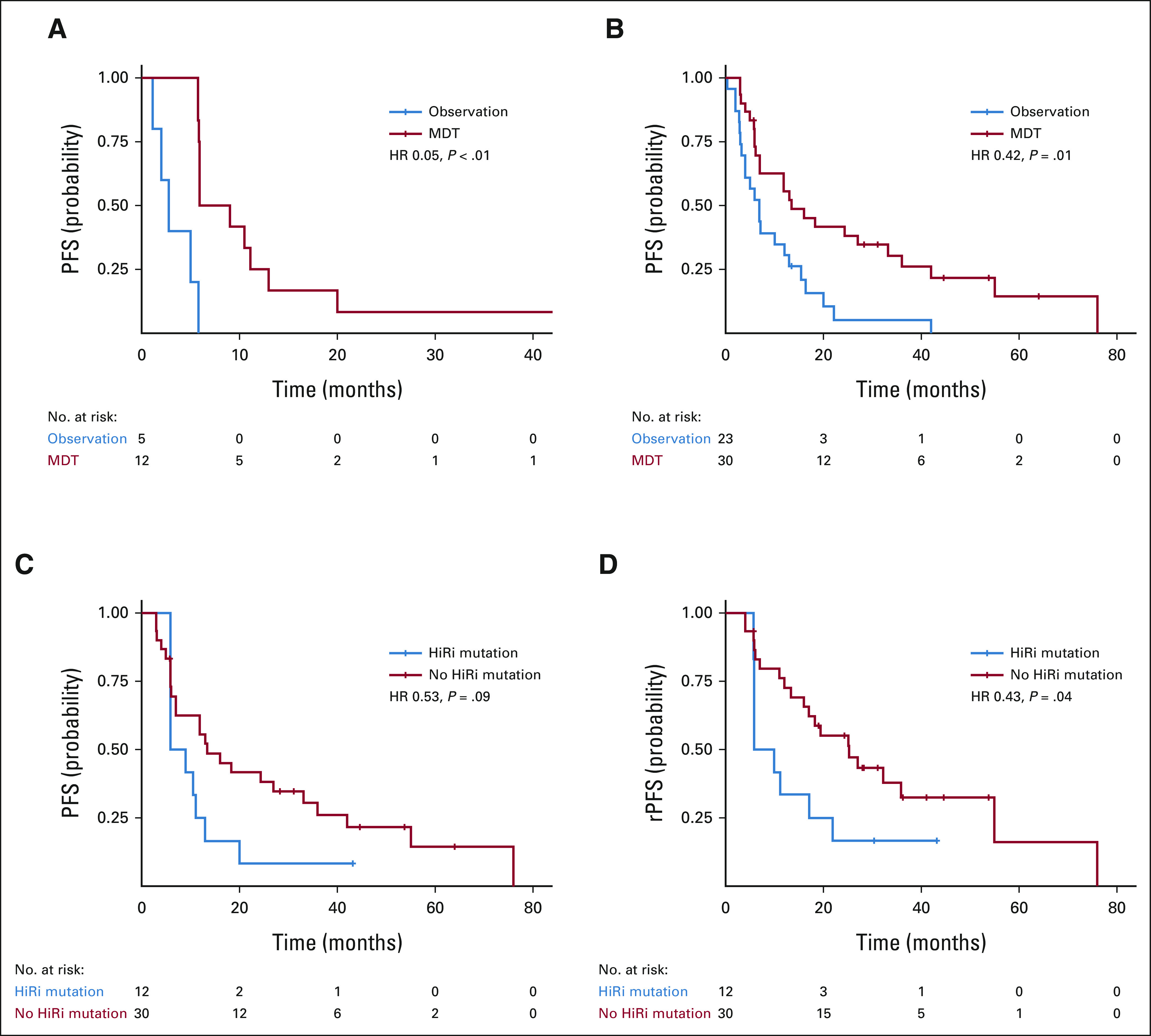
PFS stratified by treatment arm for those (A) with and (B) without a high-risk mutation stratified by treatment arm. MDT resulted in improvements in PFS in those both with and without a high-risk mutation, however, with a potential differential benefit resulting in relatively larger improvements in PFS in those with a high-risk mutation treated with MDT. (C) PFS and (D) rPFS in those treated with MDT stratified by high-risk mutation status. High-risk mutational status was prognostic for both PFS and rPFS in those treated with MDT, with longer times to events in those without a high-risk mutation. HiRi, high-risk; MDT, metastasis-directed therapy; OS, overall survival; PFS, progression-free survival; rPFS, radiographic progression-free survival.

We then stratified patients by both treatment arms and separately on the basis of high-risk mutational status to assess differential magnitude of benefit of MDT. Both those with and without a high-risk mutation benefited from MDT; however, a potential larger magnitude of benefit was experienced in those with a high-risk mutation. Tumors harboring a high-risk mutation treated with MDT experienced a median PFS of 7.5 months (95% CI, 5.9 to not reached [NR]) compared with a PFS of 2.8 months (95% CI, 2 to NR) with observation (HR, 0.05; 95% CI, 0.01 to 0.28; *P* < .01, Fig 3A). In tumors without a high-risk mutation, the median PFS with MDT was 13.4 months (95% CI, 7.0 to 36) compared with 7.0 months (95% CI, 4.0 to 15.4) with observation (HR, 0.42; 95% CI, 0.23 to 0.77; *P* = .01, Fig 3B) with a p-interaction of 0.12 (Data Supplement). Differences in rPFS were not seen (high-risk mutation: HR, 0.83; *P* = .74; no high-risk mutation: HR, 0.82, *P* = .58; *P* interaction: .40).

Within the MDT cohort alone (Fig 3C), the PFS was 13.4 months (95% CI, 7.0 to 36.0) without a high-risk mutation, compared with 7.5 months (95% CI, 5.9 to NR) with a high-risk mutation (HR, 0.53; 95% CI, 0.25 to 1.11; *P* = .09). The median rPFS after MDT was 25.3 months (95% CI, 17.0 to NR) without a high-risk mutation, compared with 8.0 months (95% CI, 5.9 to NR) with a high-risk mutation (HR, 0.43; 95% CI, 0.20 to 0.95; *P* = .04; Fig 3D).

## DISCUSSION

MDT is rapidly emerging as a therapy in omCSPC, and this study presents long-term outcomes and genomic predictors of response to MDT in omCSPC. We report that with long-term follow-up, STOMP and ORIOLE MDT remains associated with improved PFS. Of note, the PFS beyond four years was 15%-20% with MDT regardless of mutation status, and thus, a sizable proportion of patients will experience durable response to therapy. Although more follow-up is needed, the encouraging PFS report here suggests that in appropriately selected patients, MDT without systemic therapy might be a reasonable option upfront in well-informed patients wishing to avoid side effects of androgen deprivation. However, future trials, which are planned or underway, will more rigorously study this question.

In the quest for treatment personalization in omCSPC,^10,11^ genetic biomarkers are likely to play a critical role.^3,4,12-15^ Within our cohort, those treated with MDT without a high-risk mutation experienced the best outcomes (median PFS 13.4 months), whereas observation in those with a high-risk mutation experienced the poorest outcomes (median PFS 2.8 months). This suggests that individuals with omCSPC without a high-risk mutation might initially be treated with MDT alone and conversely highlights the need for novel treatment paradigms in those with a high-risk mutation. Importantly, although, those both with and without a high-risk mutation appeared to benefit from MDT, thus suggesting that this therapy should be offered to most, if not all, omCSPC. Ongoing trials combining systemic therapy (DART trial: ClinicalTrials.gov identifier: NCT04641078) or radiopharmaceuticals (RAVENS trial: ClinicalTrials.gov identifier: NCT04037358)^16^ might help define novel paradigms and hopefully further elucidate the role of genetic biomarkers within this population.

There are several limitations to this report. First, the genomic analysis did not have an a priori end point and was based on small sample size. Thus, prospective validation is needed. Second, differing imaging modalities were used (conventional in ORIOLE and choline in STOMP) and with the introduction of PSMA, how we define omCSPC might change in the future. Nevertheless, these data provide a framework to investigate such questions in the future.

In conclusion, long-term outcomes of STOMP and ORIOLE demonstrate sustained benefit to MDT over observation in omCSPC. Genomic alterations appear to have prognostic value in this patient population, suggesting that biomarkers should be evaluated in future studies to optimize patient selection.

## Data Availability

Individual deidentified participant data that underlie the results reported in this article will be shared as will the individual study protocols. The data will become available beginning 1 year and for 3 years following publication to researchers with a methodologically sound proposal to achieve aims in the previously said sound proposal. Proposals should be directed toward the corresponding authors Drs Piet Ost and Phuoc T. Tran. Data will be available in our university's data warehouse but without researcher support other than deposited metadata.
